# A sensitive serodiagnostic tool for the detection of active infection of zoonotic visceral and nasopharyngeal linguatulosis

**DOI:** 10.14202/vetworld.2019.883-889

**Published:** 2019-06-24

**Authors:** Marwa M. Attia, Elshaimaa Ismael, Nagla M. K. Saleh

**Affiliations:** 1 Department of Parasitology, Faculty of Veterinary Medicine, Cairo University, Giza 12211, Egypt; 2 Department of Veterinary Hygiene and Management, Faculty of Veterinary Medicine, Cairo University, Giza 12211, Egypt; 3 Department of Zoology, Faculty of Science, Aswan University, Aswan, Egypt

**Keywords:** halzoun, indirect enzyme-linked immunosorbent assay, *Linguatula serrata*, sandwich enzyme-linked immunosorbent assay, visceral linguatulosis

## Abstract

**Aim:**

This study aimed to evaluate the different serological techniques for early diagnosis of acute concurrent infections with linguatulosis in the definitive host (dogs) and an intermediate host (goats). This evaluation compared between the gold standard (GS) test (GS; examination of nasal and fecal samples in dogs and examination of lymph nodes in goats), sandwich enzyme-linked immunosorbent assay (S-ELISA), and indirect ELISA.

**Materials and Methods:**

Fifty goats and fifty dogs were examined for the presence of *Linguatula serrata* nymphs and adults, respectively, besides the collection of blood samples from the examined animals for serologic testing.

**Results:**

In goats; GS, S-ELISA, and indirect ELISA showed positivity in 32 (64%), 28 (56%), and 39 (78%) samples, respectively. In dogs; GS, S-ELISA, and indirect ELISA showed positivity in 25 (50%), 25 (50%), and 30 (60%) samples, respectively. S-ELISA displayed significant higher agreement with the GS test (≥0.83) than indirect ELISA (≤0.67) in both hosts. Infection with linguatulosis showed significant relation with the age of goats and dogs and the sex of goats (p<0.05).

**Conclusion:**

S-ELISA displayed more sensitivity and specificity for the detection of concurrent infections with linguatulosis in both dogs and goats than indirect ELISA, which could detect the prior infections. Similarly, these assays could be used for diagnosis of concurrent infections with linguatulosis in human, especially the chronic ones.

## Introduction

*Linguatula serrata* is a cosmopolitan worldwide aberrant endoparasite of the class *Pentastomida* [1]. The life cycle of *L. serrata* involves definitive host (dogs, cats, foxes, and other carnivores) and intermediate hosts (herbivorous animals). Infection mechanism is by eggs ingestion; these eggs contaminate the soil and the grass through infected nasal secretions and feces.

*L. serrata* adults are worm-like a parasite of the upper respiratory system, including the nasal cavities, turbinate, and rarely frontal sinuses of carnivorous animals as natural definitive hosts [2]. The eggs are expelled from the respiratory passage of the final host; therefore, contaminate pastures and water resources. When the infected eggs are swallowed by suitable herbivorous animals, the larvae reach the mesenteric lymph nodes (MLNs), liver, lungs, spleen, rarely the eyes, and other organs.

Different epidemiological studies were recorded in many countries throughout the world as in Egypt [2-6] and Iran [7-13]. Villedieu *et al*. [14] recorded infection in dogs in the United Kingdom. Different surveys were recorded in Turkey [15-17], while Gomez-Puerta *et al*. [18] recorded the first finding of the nymphal stage in a (vicuna) in Peru. Occurrence in dogs and livestock was recently recorded in Bangladesh and Australia [19,20].

*L. serrata* has zoonotic importance for human. Many cases were reported in different areas worldwide from which two patients were diagnosed with liver pentastomiasis associated with rectal adenocarcinoma [21-23]. Several cases recorded with nasopharyngeal infection or halzoun (marrara syndrome) [24-26]. Ocular linguatulosis was reported [27,28], in which the *L. serrata* nymphs attack and damage the eyes.

Different pathological changes were detected in infected lymph nodes as softening, hemorrhages, and necrosis. Histopathologic findings revealed severe infiltration of leukocytes, macrophage, and eosinophilia with necrosis and loss of morphologic features of lymphoid follicles and its trabeculae [19,29].

The morphological features of nymphs and adult males and females of *L. serrata* were described as; tongue-shaped, flattened dorsoventrally, and annulated body containing about 80-90 segments. The broad anterior end is composed of the oral opening which is subterminal and squarish, with two pairs of compound hooks surrounding this oral opening. Each body segment is covered with minute spines. The posterior part ends with the anal opening [2,30].

Although different literatures described in detail the prevalence and morphology of *L. serrata* nymphs and adults; few reports described the serologic techniques used for diagnosis of linguatulosis except two reports in which one used the counter immune-electrophoresis as tool for the diagnosis [31], and the other report used the indirect enzyme-linked immunosorbent assay (ELISA) on sheep and goats in Greece [32]. Moreover, there were no reports on the diagnosis of concurrent linguatulosis using antigens prepared from nymphs and adults of *L. serrata* collected from infected goats and dogs.

This study aimed for evaluation of the potential use of *L. serrata* somatic antigens for the early detection of acute linguatulosis in goats and dogs, with a comparison between the postmortem examination (the gold standard [GS] test) and different serologic techniques (sandwich ELISA [S-ELISA] and indirect ELISA).

## Materials and Methods

### Ethical approval

All study procedures were approved by the Institutional Animal Care and Use of Ethical Committee of the Faculty of Veterinary Medicine, Cairo University, Egypt.

### Sampling and GS test

#### In goats

Fifty slaughtered goats from butcher shops were inspected from March 2018 to August 2018. Blood samples for serum were collected from each animal, and MLNs were obtained and grossly examined by naked eyes for detection of *L*. *serrata* nymphs, according to Attia *et al*. [2] and Sinclair [33], and placed in warm saline solution 0.9% at 37°C for 20 min. Then, the saline solution was examined under a stereoscopic microscope (LEICA M60, United State).

#### In dogs

Blood, fecal, and nasal swabs were collected from 50 dogs from different governorates from March 2018 to August 2018. A positive control case was recorded in the hospital of Faculty of Veterinary Medicine, Cairo University; which sneezed *L. serrata* adult parasites from its nasal cavity. All were dogs examined for the presence of *L. serrata* eggs in feces and nasal swabs, according to Attia *et al*. [2].

### Identification of nymphs and adult of *L. serrata*

The collected nymphs and adults were washed in saline solution, relaxed, flattened, fixed, stained, mounted, and identified, according to Attia *et al*. [2] and Razavi *et al*. [34].

### Serological diagnosis

#### Preparation of larval antigens

The nymphs and adults were washed several times with saline solution. Antigens were prepared from 500 nymphs and ten adults recovered from dogs. Antigens were preserved in phosphate-buffered saline (PBS) (pH 7.2) and prepared [32]. The protein content of the two antigens was measured according to Lowry *et al*. [35].

#### Preparation of hyperimmune sera

Six male rats (*Rattus norvegicus albino*) about 150 g in weight were raised for preparation of hyperimmune sera versus *L. serrata* nymphs and adults according to Innocenti *et al*. [36], with some modification. The rats housed in two groups (two negative controls; two immunized against *L. serrata* nymphs; and two immunized against *L. serrata* adults) in conventional rat cages. The rats were supplied with balanced rats’ pellets and water *ad libitum*. The immunized rats were subcutaneously injected with 1 mg protein of antigens, which mixed in 1 ml mineral oil (1^st^ dose). Then, two subsequent intramuscular injections of 0.5 mg protein antigens mixed in the same volume of mineral oil were injected each 2 weeks interval. Rats were slaughtered for collection of blood for sera, 2 weeks after the last dose. The collected hyperimmune rats’ sera were stored at −20°C until used.

#### S-ELISA and indirect ELISA

All serum samples collected from the examined goats and dogs were tested with S-ELISA and indirect ELISA. Hyper-immunized rat sera against *L. serrata* nymphs and adults; negative control sera from rats and 1-month old goats and puppies, and positive control sera from naturally infected goats and dogs with *L. serrata* were used in each test.

#### Cross-reaction of the two tests with *L. serrata* antibodies

In addition to the sera from *L. serrata* cases, sera from dogs infected with *Toxocara canis* and *Echinococcus granulosus* adults were collected. Likewise, sera from goats infected with *Haemonchus contortus* adult and hydatid cyst were collected.

### Indirect ELISA

The checkerboard titration for determination of the lowest antigen concentration and sera dilution was done, according to Harlow and Lane [37]. A ninety-six well, flat-bottomed ELISA plates were incubated overnight with 100 μl/well of antigen at 20 μg/ml for nymphs and 10 μg/ml coating buffer (pH 9.6) for an adult in 4°C. 200 μl/well of the blocking buffer (Bovine serum albumin - PBS) was added for 2 h at 37°C. After 3 times washings; 100 μl of diluted sera 1:100 in PBS were incubated at 37°C for 2 h. 100 μl/well of horseradish peroxidase anti-goat and anti-dogs IgG; anti-rat IgG conjugate (Sigma, A-5420) diluted at 1:1000 were added to each well and incubated for 1 h at room temperature. A 100 µl of a substrate buffer contains (10 mg Ortho-Phenylenediamine) in citrate buffer and 30% H_2_O_2_ were added to the plate wells. Finally, the ELISA plate was stopped by adding 3N H_2_SO_4_, and the absorbance values or optical density (OD) were obtained from an ELISA reader (Bio-Rad, USA) at 450 nm. The sera were considered to be positive when the absorbance values were as or more than the cut off value equal to a double fold of the mean negative sera; the ELISA was done, according to Kouam *et al*. [32].

### S-ELISA

Checkerboard titration was done according to Harlow and Lane [37] to obtain the different dilution of antigen, sera, and conjugate. A ninety-six well, flat-bottomed ELISA plates were incubated overnight with 100 μl/well of hyperimmune sera from adult and nymphs diluted in carbonate buffer. After 3 times washings with PBS- Tween-20; 100 μl of diluted antigens of nymphs and adults at 10 μg/ml in PBS were incubated 37°C for 2 h. 100 μl/well of naturally infected and unknown sera were added with concentration 1:100 in PBS. Then, run the ELISA plate as indirect ELISA procedure mentioned above. The procedure followed according to Ana and Finlay [38], Rui *et al*. [39], and Dixit *et al*. [40].

### Statistical analysis

The cutoff point for a positive result was taken as 2× mean of OD of negative control sera [41]. To evaluate the values of sensitivity, specificity, accuracy, positive predictive values (PPV), and negative predictive values (NPV) and other points of screening diagnostic, individual screening tests were performed in ELISA, S-ELISA, and GS tests. The GS was considered the most sensitive and was assigned as a benchmark test.

Statistical analysis of agreement between ELISA, S-ELISA, and GS tests was performed using the Kappa agreement test. The Landis and Koch scale [42] was used to measure the degree of agreement according to the Kappa value, with the scores divided into: <0 no agreement; 0.0-0.20 slight; 0.21-0.40 fair; 0.41-0.60 moderate; 0.61-0.80 substantial; 0.81-0.99 almost perfect; and 1 perfect. Pearson correlation was estimated between ELISA, S-ELISA, and GS tests. Differences between groups were assessed by Chi-square test. Values of p≤0.05 were considered statistically significant. All statistical inference was performed on PASW Statistics, Version 18.0. software (SPSS Inc., Chicago, IL, USA).

## Results and Discussion

### Identification of the parasites

**Figure-1:**
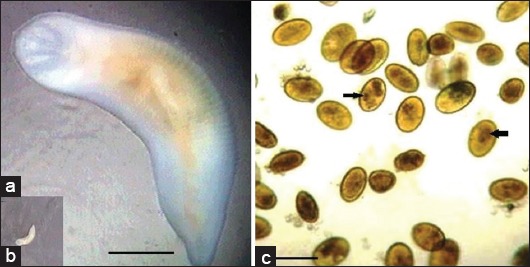
Adult of *Linguatula serrata* (a and b) showing the anterior end of the adult with four hooks surround mouth openings. (b) The eggs of *L. serrata* from nasal swabs showing the claw of the larvae pointed by arrows. Scale bar in (a) 1 mm, while in (c) 100 μm.

The nymphs, adults, and its eggs identified for *L. serrata* as recorded by Attia *et al*. [2] (Figure-1).

### Characterization of antigens

The *L. serrata* crude extract antigens were found to contain 20 and 30 μg/ml of total protein in nymphs and adults, respectively.

### Checker board titration

In indirect ELISA; the concentration of the antigens were 20 μg and 10 μg/ml for nymphs and adult respectively, and the diluted serum was 1:100. While in S- ELISA; the concentration of the antigens were 10 μg/ml for nymphs and adults, and the diluted serum was 1:100, while the conjugate for both tests was 1:1000.

### Cross-reaction of the two tests with *L. serrata* antibodies

Crude antigens of *T. canis* adult and *E. granulosus* adults, *H. contortus* adult, and *E. granulosus* hydatid cyst scolices were used and processed for specificity analyses. There was no cross-reaction recorded in the two tests, all the OD values for those crude antigens positive sera were <0.25, whereas the OD value of control negative sera of rats was 0.12 and of dogs was 0.23 while in goats were 0.20.

### OD of positive control sera from dogs and goats

The cutoff values were 0.51 and 0.56 for dogs’ sera and 0.51 and 0.50 for goats’ sera while using indirect ELISA and S-ELISA, respectively. Positive OD values for dogs ranged between 0.5 and 1.0 in indirect ELISA while in S-ELISA the OD values were 0.6-1.9. The OD values in goats were 0.45-1.8 in indirect ELISA while in S-ELISA the OD values in positive cases recorded 0.5-1.8.

### Positive indirect ELISA, S-ELISA, and GS tests in goats

Examination of lymph nodes in goats (GS) showed positivity in 32 samples (64%). The indirect ELISA test of goats’ sera showed seropositivity in 39 samples (78%) while the S-ELISA showed seropositivity in 28 samples (56%) (Table-1).

**Table-1:** ELISA, S-ELISA, and GS result in samples from goats and dogs, Cairo, Egypt, 2018 (n=50 goats; 50 dogs).

Diagnostic test	GS (%)	S-ELISA (%)	Indirect ELISA (%)
Goats			
Positive	32 (64)	28 (56)	39 (78)
Negative	18 (36)	22 (44)	11 (22)
Dogs			
Positive	25 (50)	25 (50)	30 (60)
Negative	25 (50)	25 (50)	20 (40)

ELISA=Enzyme-linked immunosorbent assay, S-ELISA=Sandwich enzyme-linked immunosorbent assay, GS=Gold standard (examination of lymph nodes in goats and examination of feces in dogs)

The indirect ELISA test of goats’ sera showed a sensitivity of 100%, and specificity of 61.11%, with an accuracy of 86% while the S- ELISA test showed sensitivity of 87.5%, and specificity of 100%, with an accuracy of 92% (Table-2).

**Table-2:** Screening test results for indirect ELISA and S-ELISA in a sample from goats and dogs, Cairo, Egypt, 2018.

Screening test	Goats		Dogs	
	
	Indirect-ELISA (%)	S-ELISA (%)	Indirect-ELISA (%)	S-ELISA (%)
Sensitivity	100	87.5	84	96
Specificity	61.11	100	64	96
Type 1 error (false positive)	38.89	0	36	4
Type 2 error (false negative)	0	12.5	16	4
Apparent prevalence	78	56	60	50
PPV	82.05	100	70	96
NPV	100	81.82	80	96
Accuracy	86	92	74	96
Likelihood ratio positive	2.57	-	2.33	24
Likelihood ratio negative	0	0.125	0.25	0.04

Indirect-ELISA=Indirect enzyme-linked immunosorbent assay, S-ELISA=Sandwich enzyme-linked immunosorbent assay, PPV=Positive predictive value, NPV=Negative predictive value, GS=Gold standard; True prevalence determined by GS was 64% in goats and 50% in dogs

For the agreement analysis, the Kappa values obtained were significantly substantial between ELISA and GS tests (K=0.67; p=0.000) and between S-ELISA and GS (K=0.83; p=0.000), while it was significantly moderate between ELISA and S-ELISA (K=0.53; p=0.000) (Table-3).

**Table-3:** Agreement between indirect ELISA, S-ELISA, and GS in samples from goats and dogs, Cairo, Egypt, 2018.

Animal	Diagnostic test	Kappa value	Agreement	p-value
Goats	Indirect-ELISA versus GS	0.67	Substantial	0.000
	S-ELISA versus GS	0.83	Substantial	0.000
	Indirect-ELISA versus S-ELISA	0.53	Moderate	0.000
Dogs	Indirect-ELISA versus GS	0.48	Moderate	0.001
	S-ELISA versus GS	0.92	Almost perfect	0.000
	Indirect-ELISA versus S-ELISA	0.48	Moderate	0.001

Indirect-ELISA=Indirect enzyme-linked immunosorbent assay, S-ELISA=Sandwich enzyme-linked immunosorbent assay, GS=Gold standard (examination of lymph nodes in goats and examination of feces in dogs)

Using the Pearson’s test for correlation, a significant positive correlation was proved between the number of nymphs detected by GS test and the optical densities obtained from indirect ELISA and S-ELISA tests (r=0.85 and r=0.90; p<0.000; respectively) (Table-4).

**Table-4:** Correlation between indirect ELISA, S-ELISA, and GS in samples from goats and dogs, Cairo, Egypt, 2018.

Animal	Diagnostic test	Pearson correlation	p-value
Goats	Indirect-ELISA versus GS	0.85	0.000
	S-ELISA versus GS	0.90	0.000
	Indirect-ELISA versus S-ELISA	0.93	0.000
Dogs	Indirect-ELISA versus GS	-	-
	S-ELISA versus GS	-	-
	Indirect-ELISA versus S-ELISA	0.69	0.000

Indirect-ELISA=Indirect enzyme-linked immunosorbent assay, S-ELISA=Sandwich enzyme-linked immunosorbent assay, GS=Gold standard (examination of lymph nodes in goats and examination of feces in dogs)

### Positive indirect ELISA, S-ELISA, and GS tests in dogs

The examination of feces in dogs (GS) showed positivity in 25 samples (50%). The indirect ELISA test of dogs’ sera showed seropositivity in 30 samples (60%), while the S-ELISA showed seropositivity in 25 samples (50%) (Table-1).

The indirect ELISA test of dogs’ sera showed sensitivity of 84% and specificity of 64%, with an accuracy of 74%, while the S-ELISA test showed sensitivity of 96% and specificity of 96%, with an accuracy of 96% (Table-2).

For the agreement analysis, the Kappa value obtained was significantly moderate between ELISA and GS tests (K=0.48; p=0.001), and between ELISA and S-ELISA (K=0.48; p=0.001), while it was significantly almost perfect between S-ELISA and GS (K=0.92; p=0.000) (Table-3).

### Frequency of infection in goats and dogs

**Figure-2:**
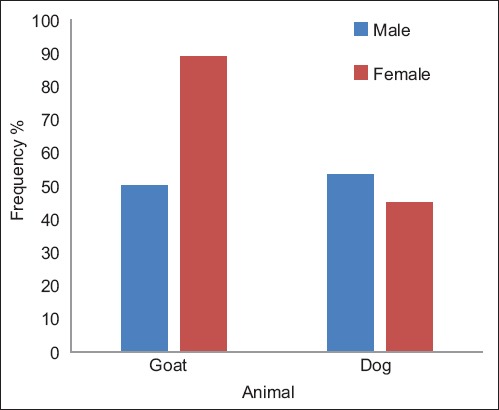
Frequency of *Linguatula serrata* infection in goats and dogs in relation to sex of the animal, Cairo, Egypt, 2018.

In goats, the frequency of *L. serrata* infection was related to the sex of the animal and was significantly higher in female goats than in males (p=0.006). However, in dogs, the frequency of infection was not statistically related to the sex of the animal (p>0.05) (Figure-2, Table-5).

**Figure-3:**
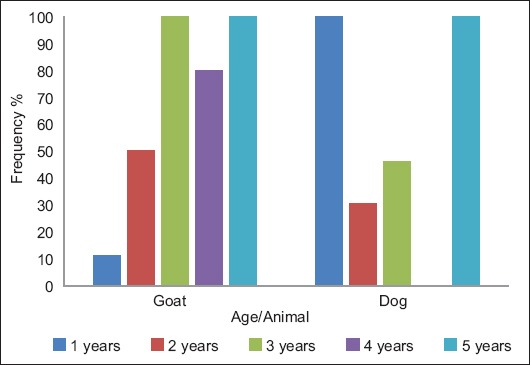
Frequency of *Linguatula serrata* infection in goats and dogs in relation to the age of the animal, Cairo, Egypt, 2018.

In goats, the frequency of *L. serrata* infection was related to the age of the animal and was significantly higher in goats aged 3 years or more (p=0.000). In addition, in dogs, it was statistically related to the age of the animal. Dogs of 1 year old and 5 years old showed the highest frequencies of infection (p=0.000), (Figure-3 and Table-5).

**Table-5:** Frequency of *L. serrata* infection in goats and dogs in relation to sex and age of animal, Cairo, Egypt, 2018.

Characteristics	Goat	Dog
Sex		
Female	16/18 (88.89%)	9/20 (45%)
Male	16/32 (50%)	16/30 (53.33%)
p-value	0.006*	0.387
Age (years)		
1	1/9 (11.11%)	6/6 (100%)
2	9/18 (50%)	7/23 (30.43)
3	13/13 (100%)	6/13 (46.15%)
4	4/5 (80%)	0/2 (0%)
5	5/5 (100%)	6/6 (100%)
p-value	0.000*	0.000*

*L. serrata*=*Linguatula serrata*.

* Indicate significance at p<0.05

In goats, the GS test revealed 64% positivity, while S-ELISA revealed 56% positivity in comparison to indirect ELISA which revealed 78% positivity; this is due to that S-ELISA could detect the concurrent infection, while indirect ELISA could detect the antibody levels of the previous infection from 6 months ago. Positive S-ELISA results were higher than GS, which may be due to the presence of infection in different organs other than MLNs.

From these comparisons, it was revealed that the sensitivity of the two tests was similar in goats while high in S-ELISA in dogs. The specificity of S-ELISA was high in dogs and goats.

This sensitive S-ELISA improves and simplifies the detection of linguatulosis in animals.

Pentastomiasis has a zoonotic potential to human, which is difficult to be diagnosed or might be misdiagnosed with other chronic diseases such as malignant tumors, cysticercosis, and lung diseases [43].

Advanced technique as polymerase chain reaction test was previously used for identification but had not been evaluated for diagnosis of linguatulosis [44]. For this reason, this work developed a new sensitive technique for diagnosis of concurrent infection of one of the pentastomes (*L. serrata*), and we used two animals; the definitive host (dogs) and an intermediate host (goats) that represented the most grazing animal around the dogs, to be a good example for human testing.

Using the crude extract of *L. serrata* antigens was presented a reliable, non-invasive diagnostic tool for detecting active infections. The sensitivity and specificity of any immunoassay are dependent on the antigen capture efficiency of an antibody which is subsequently governed by the nature of antibody. This led to the different assay sensitivities of the commercially available kits using different types of antibodies which could be monoclonal or polyclonal at the capture stage in ELISA.

Considering linguatulosis importance in human and animals, it seems necessary to do more investigations in both domestic and wild carnivores and herbivores. Only a few reports described the seroprevalence and evaluated the indirect ELISA for 121 sheep sera and determined its sensitivity, specificity, PPV, NPV, and accuracy of the somatic antigens, which were 90.4%, 75.0%, 84.6%, 83.7%, and 84.2%, and those for excretory-secretory antigen were 89.0%, 93.8%, 95.6%, 84.9%, and 90.9%, respectively [44]. However, indirect ELISA and S-ELISA in our study showed higher values than that report. Another report only used indirect ELISA for seroprevalence [32].

Other serological tests were used as counter immunoelectrophoresis used for diagnosis of linguatulosis in sheep [31], but that was a qualitative serological test, while the two tests used in this study were quantitative tests with high sensitivity and specificity and can be used for field studies or for human cases suffering from chronic disease, which could infect several organs.

## Conclusion

In this study, we differentiated between two assays to distinguish the sensitivity and specificity of each test. S-ELISA showed higher sensitivity and specificity than indirect ELISA, so, it can be applied in the field studies or laboratories for diagnosing of linguatulosis because it detected the concurrent circulating antigens. S-ELISA could be considered as a new serological technique for the diagnosis of linguatulosis in goats and dogs. Moreover, S-ELISA could be used in the diagnosis of linguatulosis in human with halzoun or visceral form.

## Authors’ Contributions

MMA, EI, and NMKS conceived and designed the experiments. MMA and NMKS performed the experiments. EI analyzed and interpreted the data. All authors participated in draft and revision of the manuscript. All author have read and approved the final manuscript.
